# Nutritional status of children and adolescents in three Serbian enclaves in Kosovo and Metohija

**DOI:** 10.1186/s12889-021-10848-z

**Published:** 2021-04-24

**Authors:** Valentina Fabiano, Lucia Barcellini, Marco Ugo Andrea Sartorio, Erica Pendezza, Alessandro Leone, Fabio Meneghin, Dario Dilillo, Gian Vincenzo Zuccotti

**Affiliations:** 1 Department of Pediatrics, Vittore Buzzi Children’s Hospital, Università di Milano, 32, Via Castelvetro, 20154 Milan, Italy; 2 International Center for the Assessment of Nutritional Status (ICANS), Department of Food, Environmental and Nutritional Sciences (DeFENS), Università di Milano, Milan, Italy; 3 Neonatal Intensive Care Unit, Vittore Buzzi Children’s Hospital, Università di Milano, Milan, Italy

**Keywords:** Nutritional status, Children, Adolescents, Kosovo, Transitional countries

## Abstract

**Objective:**

To evaluate nutritional status of children and adolescents living in three Serbian enclaves in Kosovo and Metohija.

**Methods:**

We conducted an observational cross-sectional, population-based study, enrolling children and adolescents who underwent a pediatric screening performed in the three Serbian enclaves of Gračanica, Gornje Kusce and Velika Hoča in Kosovo and Metohija. Children and adolescents (5–19 years) of all ethnic groups were evaluated in one of the three free outpatient medical facilities in rural villages in Kosovo. Body weight and height were measured, height-for-age z- scores (HAZ) and BMI-for-age z-scores (BAZ) indicators were analyzed. The anthropometric indicators HAZ and BAZ distributions were compared between sex and ages using Fisher’s exact test. A two-sample Z-test for proportions was used to detect differences in individual categories of height- and BMI-for-age categories across sexes and age classes.

**Results:**

Three hundred twenty-eight children and adolescents (184 females, 56.1% and 144 males, 43.9%) aged between 5 and 19 years were enrolled in the study. 241/328 participants showed a normal linear growth; with significantly more girls (78.3%) than boys (67.4%) being in the normal category. Similarly, a significant difference in BAZ distribution between sexes was noted, with more females being in the normal BMI category compared to males (63.0% vs 50.0%, respectively). Underweight and severe underweight subjects showed a prevalence of 1.5 and 0.6%, respectively. Overweight and obesity prevalence was 19.5 and 9.1%, respectively, which was comparable to World Health Organization overweight and obesity prevalence data for Serbia.

**Conclusions:**

Prevalence of undernutrition and severe undernutrition in children and adolescents living in three Serbian enclaves in Kosovo and Metohija is small. By contrast, a tendency to an increase in overweight and obesity, especially in the male population, was noted.

## Backgrounds

Improving the nutritional status in pediatric age is considered a key strategy for reaching global development goals and reducing the non-communicable diseases (NCDs) burden [1]. WHO’s 2025 Global Targets for nutrition include, among others, a 40% reduction in the number of children under-5 who are stunted, no increase in childhood overweight and reduction of wasting to below 5% [2]. Even though several remarkable improvements in childhood nutrition have been achieved in the last decades, in low, middle-income and transitional countries undernutrition still remains a significant problem. Besides that, childhood overweight prevalence is seriously increasing [3].

Assessing the nutritional status of infants, children, and adolescents is key to enable the implementation of focused nutritional interventions, which might vary according to the specific regional and socio-political settings they are addressed to.

Recently published data about the nutritional status of infants and children from some developing or transitional countries is available, as in the case of sub-Saharan Africa or Asia [4–7]; however, data is very limited from other low-middle income countries such as Kosovo, one of the poorest countries in Europe.

In July 1999, UNICEF conducted a survey on Kosovar children aged between 0 and 5 years [8], reporting a 3.1% prevalence of acute malnutrition (weight for height < − 2 z-scores) and a 10.7% prevalence of chronic malnutrition (height for age < − 2 z-scores). Two years later, in 2001, UNICEF [9], in collaboration with Kosovo’s Institute for Public Health, found a 10% prevalence of low height-for-age in children aged 6–59 months, and a 4% prevalence of low weight-for-height.

More recently, a single study has been published addressing the nutritional status of preschool children attending nursery schools in Kosovo [10]. Authors reported 3% of stunted and 1.9% of wasted children, whilst up to 8.9% of included children resulted to be overweight and 2.3% obese. 27.3% of children resulted to be at risk of becoming overweight. To our knowledge, no data about malnutrition and overweight in older children and adolescents living in Kosovo has been published to date.

The aim of the present study is to evaluate the nutritional status of a sample of children and adolescents from three Serbian enclaves in Kosovo and Metohija,

### DOCs for KiM

DOCs for KiM (Doctors for Kosovo and Metohija) is a humanitarian project born from the collaboration between “Amici di Decani”, a not-for-profit, apolitical, non-sectarian association involved in charitable activities and cultural promotion, and the Department of Pediatrics of the Vittore Buzzi Children’s Hospital, ASST-FBF-Sacco, University of Milan. The aim of the project was to establish medical facilities in order to offer free medical screenings to the pediatric population living in rural villages in Kosovo. The project has already set up three outpatient facilities in Serbian enclaves in Kosovo, in the rural villages of Gračanica, Gornje Kusce and Velika Hoča, fully equipped to perform pediatric screenings for about 3000 infants, children and adolescents living in those areas. A group of 12 pediatricians, 2 dietitians, 2 pediatric neurologists, and 1 ophthalmologist participated in the project between 2018 and 2019.

## Materials and methods

This is a cross-sectional, population-based study involving children and adolescents who underwent a pediatric screening performed by the health personnel participating in the DOCs for KiM project. Two “health weeks” took place in November 2018 and October 2019, respectively, in the three Kosovarian villages of Gračanica, Gornje Kusce and Velika Hoča.

The opening of the three outpatient facilities offering free pediatric screenings was advertized through local broadcast, as well as through billboards in the streets a about 2 months before each of the two scheduled screening weeks. During the two “health weeks”, the health personnel performed pediatric screenings in the outpatient facilities to all infants, children and adolescents of all ethnic groups who spontaneously sought medical advice. The pediatric screening included the collection of family history and personal medical history, an anthropometric assessment (height, weight, BMI), and a complete clinical examination of organs and systems. Children and adolescents aged between 5 and 19 years were eligible to participate in the present nutritional study.

The participation criteria included: (a) being a child or an adolescent evaluated in one of the three DOCs for KiM outpatient facilities during the “health week”, (b) being aged between 5 and 19 years, and (c) parents/legal representatives expressing their consent to data collection and analysis. Anthropometric measurements were collected by the 2 trained dietitians following standard guidelines [11]. Body weight was measured with the subject in underwear or in light clothing, without shoes, and using the digital weight scale Seca 877. Measures were taken to the nearest 100 g. The weighing scale was checked for accuracy with a standard weight every 50 measurements. Height was measured using the Seca 217 vertical stadiometer. The subject was in standing position with feet together, without shoes, and with shoulders, buttocks and heels touching the vertical measuring board. Measures were taken to the nearest 0,1 cm, with the subject standing with the eyes in the Frankfort horizontal plane. Both the digital scale and the stadiometer were reliable and easy to use. The Body Mass Index (BMI) was calculated as weight (kg)/height (m)^2^.

The results of weight and height measurements of children and adolescents in the three enclaves in Kosovo were compared with the growth charts from the World Health Organization (WHO Growth Reference 2007) and Z-scores were used for the comparisons. The anthropometric indicators height-for-age z-scores (HAZ) and BMI-for-age z-scores (BAZ) were used for all children and adolescents enrolled in the study [12]. The reference cutoffs for under- and over-weight screening are shown in Table 1.

**Table 1:** World Health Organization 2007 reference cutoffs for screening of under- and over-nutrition

Parameter	World Health Organization 2007 reference
Stunting (Height-for-age)	<−2 SD
Severe stunting (Height-for-age)	<−3 SD
Underweight (BMI-for-age)	<−2 SD
Severe Underweight (BMI-for-age)	<−3 SD
Overweight (BMI-for-age)	> + 1 SD
Obesity (BMI-for-age)	> + 2 SD
Severe Obesity (BMI-for-age)	> + 3 SD

The statistical analysis was performed using STATA version 12.0 (College Station, TX: StataCorp LP). Discrete variables are reported as frequency and percentage. HAZ and BAZ distributions between sexes and age classes were compared using Fisher’s exact test. A two-sample Z-test for proportions was used to detect differences in individual categories of height- and BMI-for-age categories across sexes and age classes. A value of *p* < 0.05 was considered statistically significant.

This study was conducted according to the guidelines set out in the Declaration of Helsinki and all procedures involving research study participants were approved by the Ethics Committee of ASST-FBF-Sacco. The DOCs for KiM humanitarian mission was positively welcomed by the population and all parents who turned up to the outpatient facilities to receive medical advice for their children accepted to participate in the data collection and in the study. Verbal informed consent was obtained from all parents/legal representatives of the included subjects and was witnessed and formally recorded in agreement with ASST-FBF-Sacco Ethic Committee’s approval.

## Results

A total of 328 children and adolescents (184 females, 56.1% and 144 males, 43.9%) aged between 5 and 19 years were enrolled in the study.

**Fig. 1:**
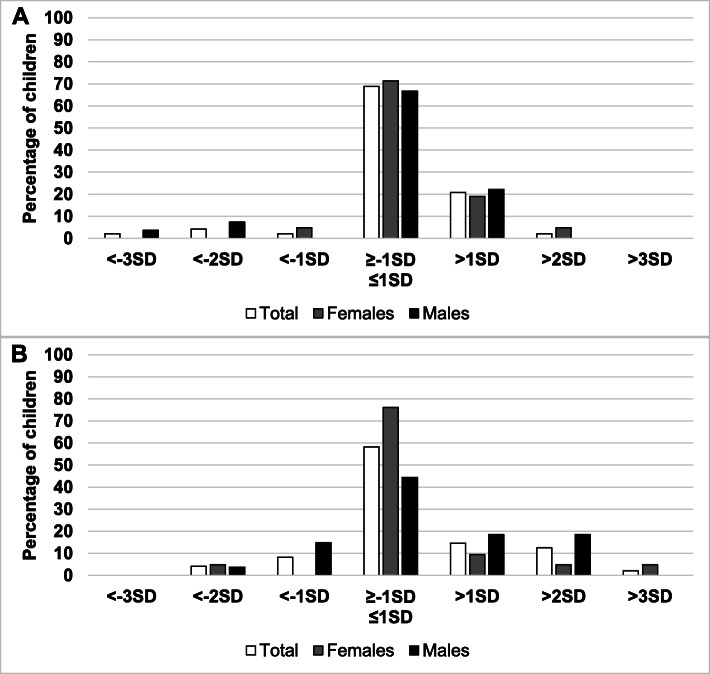
Distribution of height-for-age (**a**) and BMI-for-age (**b**) categories by sex. ^*^ = *p* < 0.05 among children (5–9 years)

**Fig. 2:**
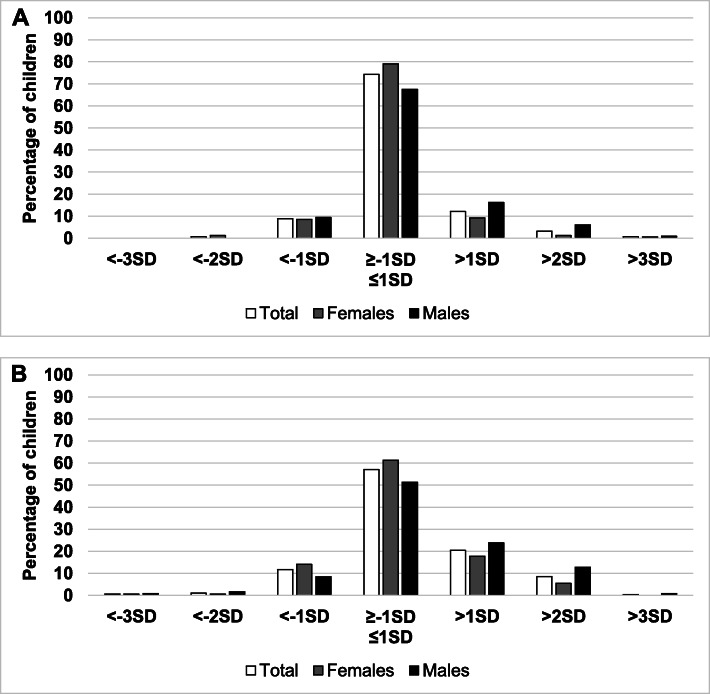
Distribution of height-for-age (**a**) and BMI-for-age (**b**) categories by sex, (^*^ = *p* < 0.05) among adolescent (10–19 years)

The overall distribution of HAZ categories by sex is reported in Fig. 1a (5–9 years) and Fig. 2a (10–19 years).

241 (73.5%) subjects (73.5%) showed a HAZ between -1SD and + 1SD, reflecting a largely normal linear growth. Sex-comparison in this HAZ category showed a statistically significant difference with more females being in the normal category compared to males (78.3% vs 67.4%, respectively). Stunting and severe stunting showed a prevalence of 1.2% and 0.3% respectively, without any difference between males and females.

The overall distribution of BAZ categories between sexes is reported in Fig. 1b (5–9 years) and Fig. 2b (10–19 years). A statistically significant difference was found in the distribution of BAZ categories by sex (*p* = 0.040). One hundred eighty-eight subjects (57.3%) showed a BAZ between -1SD and + 1SD. As for height indicators, a significant difference was noted in the BAZ distribution between sexes, with more females being in the normal BMI category compared to males (63.0% vs 50.0%, respectively). Underweight and severe underweight subjects showed a prevalence of 1.5% and 0.6% respectively, without any statistically significant difference between sexes. By contrast, the number of obese males (13.9%) was higher than obese females (5.4%) (BAZ > +2SD). The overweight and obesity prevalence in the sample was 19.5% and 9.1% respectively.

HAZ and BAZ distribution was also analyzed according to three age groups. A statistically significant difference was found in the distribution of HAZ and BAZ categories by age group (*p* = 0.001 and *p* = 0.017 respectively). Normal height (≤ − 1SD to ≥ + 1SD) showed a different distribution across age groups, with 81.3% of adolescents (15-19ys) falling in this category compared to 68.8% of children aged 5–9 years and 61% of teens aged 10–14 years, respectively. The same difference in distribution across age groups was observed with regards to BMI: normal BMI (≤ − 1SD to ≥ + 1SD) was more frequently observed in the adolescent group (15-19ys) as opposed to younger children and teens (5-9ys and 10-14ys). Obesity (BAZ > +2SD) was also differently distributed across age classes; the percentage of obese children was higher among primary school children (12.5%) and children aged 10-14ys (14.3%) compared to adolescents (5.5%). Our data did not show any significant differences in overweight (BAZ > +1SD) and severe obesity (BAZ > +3SD) distribution across age groups (Table 2).

**Table 2:** Anthropometric indicators by age

	Total		5–9 years		10–14 years		15–19 years		
	N	%	N	%	N	%	N	%	*p* value
Height-for-age									0,001
< −3SD	1	0.3	1_a_	2,1	0_a_	0	0_a_	0	
< −2SD	4	1.2	2_a_	4,2	2_a,b_	2	0_b_	0	
< −1SD	26	7.9	1_a_	2,1	11_a_	11,2	14_a_	7,7	
> = − 1SD < =1SD	241	73.5	33_a,b_	68,8	60_b_	61,2	148_a_	81,3	
> 1SD	44	13.4	10_a_	20,8	18_a_	18,4	16_b_	8,8	
> 2SD	10	3.0	1_a,b_	2,1	6_b_	6,1	3_a_	1,6	
> 3SD	2	0.6	0_a_	0	1_a_	1	1_a_	0,5	
Total	328	100.0	48	100	98	100	182	100	
BMI-for-age									0,017
< −3SD	2	0.6	0_a_	0	1_a_	1	1_a_	0,5	
< −2SD	5	1.5	2_a_	4,2	2_a,b_	2	1_b_	0,5	
< −1SD	37	11.3	4_a_	8,3	14_a_	14,3	19_a_	10,4	
> = − 1SD < =1SD	188	57.3	28_a,b_	58,3	43_b_	43,9	117_a_	64,3	
> 1SD	64	19.5	7_a_	14,6	23_a_	23,5	34_a_	18,7	
> 2SD	30	9.1	6_a,b_	12,5	14_b_	14,3	10_a_	5,5	
> 3SD	2	0.6	1_a_	2,1	1_a_	1	0_a_	0	
Total	328	100.0	48	100	98	100	182	100	

Different letters indicate statistically significant differences

The distribution of height and BMI indicators across age classes were also evaluated separately in the male and female populations. The normal height indicator (HAZ between -1SD and + 1SD) was found more frequently in female adolescents compared to younger girls (86.1% in 15-19ys vs. 71.4% in 5-9ys and 62.5% in 10-14ys), which matches the results found across the whole population. However, a statistically significant difference was only found in the 15-19ys-group compared to the 10-14ys-group. By contrast, no differences in height indicators were observed across age classes in the male group. Normal BMI (BAZ between -1SD and + 1SD) also showed a different distribution across age classes in the female group only; however, the distribution did not reflect the results found across the whole population, since the highest percentage of normal BMI was observed in the 5–9 years age class (Table 3 A-B).

**Table 3:** A-B Anthropometric indicators by age and sex (3A Female, 3B male)

A									
	Female								
	Total		5–9 years		10–14 years		15–19 years		
	N	%	N	%	N	%	N	%	*p* value
Height-for-age									0,01
< −3SD	0	0.0	0_a_	0	0_a_	0	0_a_	0	
< −2SD	2	1.1	0_a,b_	0	2_b_	4,2	0_a_	0	
< −1SD	15	8.2	1_a_	4,8	6_a_	12,5	8_a_	7	
> = − 1SD < =1SD	144	78.3	15_a,b_	71,4	30_b_	62,5	99_a_	86,1	
> 1SD	19	10.3	4_a_	19	8_a_	16,7	7_b_	6,1	
> 2SD	3	1.6	1_a_	4,8	1_a_	2,1	1_a_	0,9	
> 3SD	1	0.5	0_a_	0	1_a_	2,1	0_a_	0	
Total	184	100.0	21	100	48	100	115	100	
BMI-for-age									0,005
< −3SD	1	0.5	0_a_	0	1_a_	2,1	0_a_	0	
< −2SD	2	1.1	1_a_	4,8	1_a,b_	2,1	0_b_	0	
< −1SD	23	12.5	0_a_	0	10_b_	20,8	13_a,b_	11,3	
> = − 1SD < =1SD	116	63.0	16_a_	76,2	21_b_	43,8	79_a_	68,7	
> 1SD	31	16.8	2_a_	9,5	11_a_	22,9	18_a_	15,7	
> 2SD	10	5.4	1_a_	4,8	4_a_	8,3	5_a_	4,3	
> 3SD	1	0.5	1_a_	4,8	0_a,b_	0	0_b_	0	
Total	184	100.0	21	100	48	100	115	100	
B									
	Male								
	Total		5–9 years		10–14 years		15–19 years		
	N	%	N	%	N	%	N	%	*p* value
Height-for-age									0,047
< −3SD	1	0.7	1_a_	3.7	0_a_	0.0	0_a_	0.0	
< −2SD	2	1.4	2_a_	7.4	0_a,b_	0.0	0_b_	0.0	
< −1SD	11	7.6	0_a_	0.0	5_a_	10.0	6_a_	9.0	
> = − 1SD < =1SD	97	67.4	18_a_	66.7	30_a_	60.0	49_a_	73.1	
> 1SD	25	17.4	6_a_	22.2	10_a_	20.0	9_a_	13.4	
> 2SD	7	4.9	0_a_	0.0	5_a_	10.0	2_a_	3.0	
> 3SD	1	0.7	0_a_	0.0	0_a_	0.0	1_a_	1.5	
Total	144	100.0	27	100.0	50	100.0	67	100.0	
BMI-for-age									0,537
< −3SD	1	0.7	0_a_	0.0	0_a_	0.0	1_a_	1.5	
< −2SD	3	2.1	1_a_	3.7	1_a_	2.0	1_a_	1.5	
< −1SD	14	9.7	4_a_	14.8	4_a_	8.0	6_a_	9.0	
> = − 1SD < =1SD	72	50.0	12_a_	44.4	22_a_	44.0	38_a_	56.7	
> 1SD	33	22.9	5_a_	18.5	12_a_	24.0	16_a_	23.9	
> 2SD	20	13.9	5_a,b_	18.5	10_b_	20.0	5_a_	7.5	
> 3SD	1	0.7	0_a_	0.0	1_a_	2.0	0_a_	0.0	
Total	144	100.0	27	100.0	50	100.0	67	100.0	

Different letters indicate statistically significant differences

However, no conclusion should be drawn based on these observations given the limited number of subjects included in each group.

**Fig. 3:**
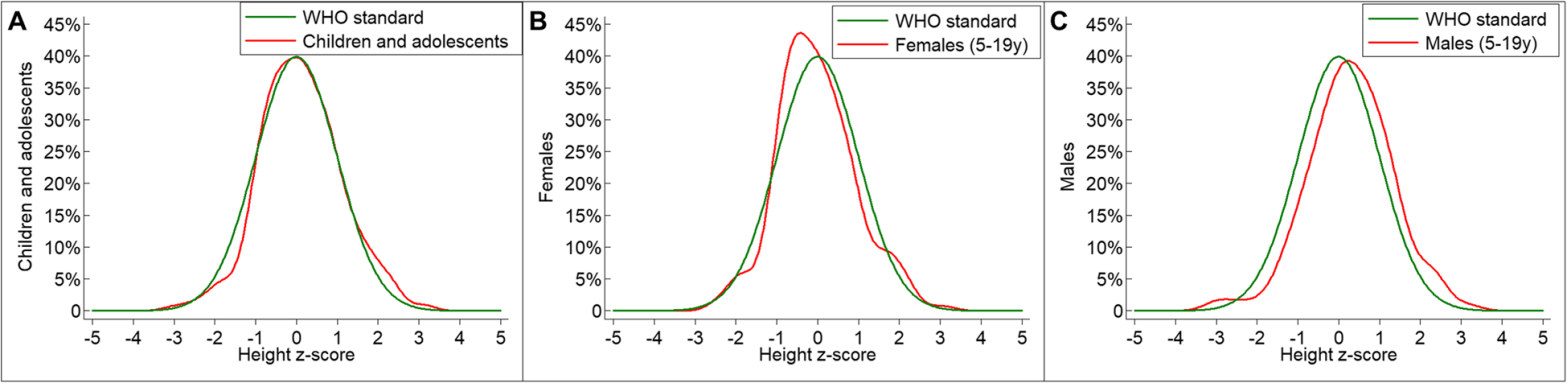
Distribution of HAZ compared to WHO Growth reference in **a** all children and adolescents, **b** females (5-19 years) and **c** males (5-19 years)

The distribution of HAZ across children and adolescents in our study was comparable to the WHO Growth reference (Fig. 3a-b-c).

**Fig. 4:**
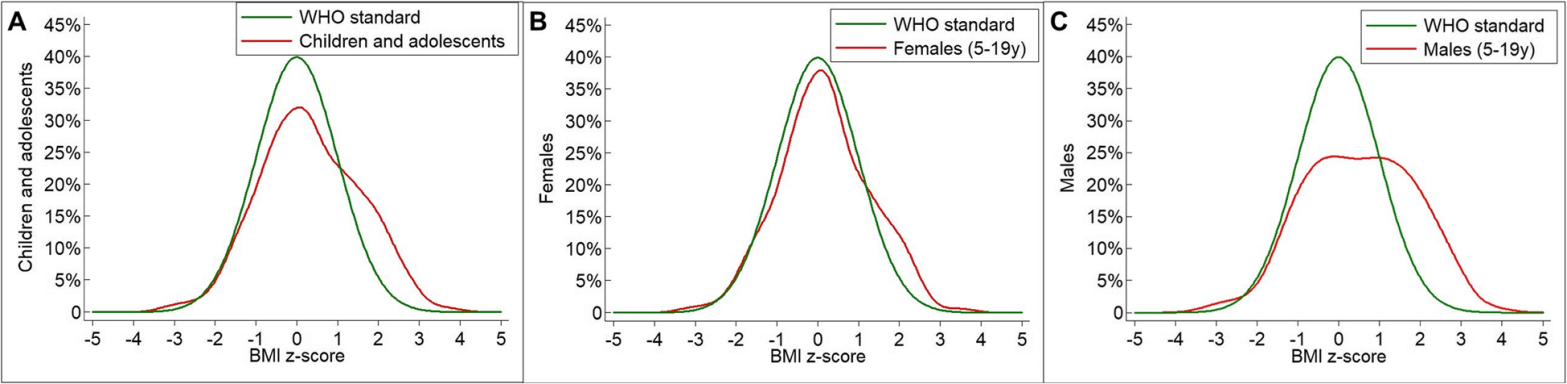
Distribution of BAZ compared to WHO Growth reference in **a** all children and adolescents, **b** females (5-19 years) and **c** males (5-19 years)

The distribution of BAZ across the whole population and across females was comparable to the WHO Growth reference (Fig. 4a-b) as well.

In the male group, our subjects showed a higher tendency to overweight compared to the WHO Growth reference (Fig. 4c).

The total percentage of our subjects showing BAZ > +2SD (9.1%) was not significantly different from the WHO obesity prevalence data for Serbia (9,8%; *p* = 0.97). The same result was also observed in relation to obesity prevalence by sex-category, which was lower in females than in males and matches the WHO obesity prevalence data for Serbia (F: *p* = 0.85; M: *p* = 0.42) [13].

## Discussion

This study evaluated the nutritional status of children and adolescents from three Serbian enclaves in Kosovo through the collection of anthropometric measurements, performed during the two DOCs for KiM humanitarian missions.

Our data demonstrated that the prevalence of stunting and underweight (1.2 and 1.5% respectively) across children and adolescents aged 5 to 19 years is generally low. This finding is in contrast with older UNICEF data [8, 9] which showed a 3.1% prevalence of acute malnutrition and a 10.7% prevalence of stunting across children aged 6–59 months. It is reasonable to assume that the Kosovar infants and children nutritional status, as described by UNICEF, might have been poorer since the UNICEF data was collected 20 years ago, at the time of the Kosovo War coming to its end. By contrast, 1,2% stunting and 1,5% underweight subjects were found in our study, which suggests that malnutrition might be less prevalent nowadays. The undernutrition prevalence in our study was also lower than the 3% prevalence of stunting from a study performed in Kosovo in 2017 [10]. Although Kosovo remains one of the poorest countries in Europe, it has undergone a slow though progressive transition to better living conditions. The anthropometric data of the present study might suggest an improvement in the nutritional status in pediatric age compared to previously published data. However, we cannot exclude that our observations might be based on an underestimate, given the reduced sample size. Moreover, in drawing comparisons, it is important to consider that both the UNICEF data [8, 9] and those by Rysha et al. were collected in infants and children aged under 5, whilst the data from the present study concerned older children and adolescents. Nevertheless, a better nutritional status in older children and adolescents might be considered as a consequence of a better nutritional status since infancy and early childhood. In fact, we should also consider that the anthropometric measurements of the present study were collected in a sample of subjects who spontaneously turned up to the outpatient facilities, therefore, our observations might be the result of a selection bias, since the families who decided to participate in the data collection might have been the ones with a better awareness of the importance of prevention and medical screening.

In our population, a different distribution of normal height and weight was observed based on age. In both instances, the percentages of normal height (HAZ between -1SD and + 1SD) and of normal BMI (BAZ between -1SD and + 1SD) were higher in the adolescent group than across younger teens and children. Despite this observation, we did not find any statistically significant difference in the distribution of pathologic BMI classes, although we observed a rather clear tendency to overweight across the younger children compared to older children, teens and adolescents. The lack of statistical significance might be explained based on the small sample size of each age group.

Obesity and overweight had a 9,1% and 19,5% prevalence, respectively. The obesity prevalence in our study was higher compared to the previous data collected in younger children in Kosovo in 2017 [10], which showed an obesity prevalence of 2,3%. The prevalence of overweight was also found to be higher compared to Rysha et al. [10], who, however, operated a distinction between overweight (8,9%) and risk of being overweight (27,3%), by also referring to the 2006 WHO standards for infants and younger children. Our overweight and obesity prevalence data is in line with the overweight [14] and obesity [13] WHO prevalence data for the Serbian population aged 5–19 years.

When considering the BMI distributions according to age and sex group, normal weight was found to be significantly more common in females than in males. In fact, the BAZ distribution curve of our male patients is significantly more flat than the WHO reference curve, which reflects the lower percentage of male being in the normal BAZ category, and, though not statistically significant, a tendency to overweight and obesity.

This study shows some limitations. Firstly, the small sample size, since the 328 enrolled subjects represent only 10% of the nearly 3000 infants, children and adolescents estimated to be living in those three enclaves. This limitation is even more evident in conducting analyses across different age groups. Another limitation is the cross-sectional study design. The subjects included in the study spontaneously turned up to the outpatient facilities, thus potentially determining a selection bias. Moreover, we exclusively collected weight and height data. It would be meaningful to evaluate other anthropometric indexes, such as body circumferences and skinfold thickness measurements. Lastly, the subjects included in the study were mainly Serbian, however, no specific data on ethnic origin was collected and therefore we were unable to draw a comparison across children and adolescents of different ethnicities.

As a future perspective, it would be interesting to compare the present data with data collected in a further nutritional study conducted on the same population.

## Conclusions

Our data demonstrated that the undernutrition (1.5%) and severe undernutrition (0.6%) prevalence in children and adolescents living in three Serbian enclaves in Kosovo and Metohija is marginal and has presumably declined over the last two decades. This is a reassuring result given that, although the country’s hardest time is over, Kosovo is still one of the poorest countries in Europe and the living conditions of infants, children and adolescents, and especially of those belonging to ethnic minorities, are still far below European standards.

By contrast, the tendency to an increase in overweight and obesity represents a worrying trend. Overweight children would benefit from receiving support since early infancy in relation to healthy weight maintenance, physical activity opportunities, and chronic disease prevention. Interventions should address the whole spectrum of malnutrition, taking into account overweight prevention as well as undernutrition.

Even though some positive trend is shown by the results of the present study, evaluating the nutritional status of infants, children and adolescents remains key, and should be addressed as a matter of priority by health policies in less developed countries. Our study is the first in this field taking into account the nutritional status in the Serbian-Kosovar pediatric population over 5 years, and provides up-to-date data collected in one of the most neglected European regions.

## Data Availability

The datasets used and/or analyzed in the current study are available from the corresponding author upon reasonable request.

## Abbreviations

HAZ: height-for-age z- scores
BMI: body mass index
BAZ: BMI-for-age z-scores
WHO: World Health Organization
